# Automatic Conversational Scene Analysis in Children with Asperger Syndrome/High-Functioning Autism and Typically Developing Peers

**DOI:** 10.1371/journal.pone.0085819

**Published:** 2014-01-29

**Authors:** Alessandro Tavano, Anna Pesarin, Vittorio Murino, Marco Cristani

**Affiliations:** 1 Institute of Psychology, University of Leipzig, Leipzig, Germany; 2 Scientific Institute “E.Medea”, San Vito al Tagliamento (Pordenone), Italy; 3 Department of Computer Science, University of Verona, Verona, Italy; 4 Italian Institute of Technology (IIT), Genova, Italy; University of Western Brittany, France

## Abstract

Individuals with Asperger syndrome/High Functioning Autism fail to spontaneously attribute mental states to the self and others, a life-long phenotypic characteristic known as mindblindness. We hypothesized that mindblindness would affect the dynamics of conversational interaction. Using generative models, in particular Gaussian mixture models and observed influence models, conversations were coded as interacting Markov processes, operating on novel speech/silence patterns, termed Steady Conversational Periods (SCPs). SCPs assume that whenever an agent's process changes state (e.g., from silence to speech), it causes a general transition of the entire conversational process, forcing inter-actant synchronization. SCPs fed into observed influence models, which captured the conversational dynamics of children and adolescents with Asperger syndrome/High Functioning Autism, and age-matched typically developing participants. Analyzing the parameters of the models by means of discriminative classifiers, the dialogs of patients were successfully distinguished from those of control participants. We conclude that meaning-free speech/silence sequences, reflecting inter-actant synchronization, at least partially encode typical and atypical conversational dynamics. This suggests a direct influence of theory of mind abilities onto basic speech initiative behavior.

## Introduction

Human beings are more aptly defined as conversational mammals, rather than simply as articulate mammals [1]. We effortlessly engage in verbal exchanges, seeking for informative as well as emotionally rewarding experiences. It has been recently acknowledged that the biological universality of such talent stands in sheer contrast with the complex set of abilities it requires, such as the tightly timed coordination of speech, facial gestures, respiratory kinematics, bodily posture, visual and auditory attention [2]–[4]. How are these different levels of behavior and sensory experience successfully integrated? Some philosophers suggest that individuals might use normative frames of reference to reach a sufficient degree of mutual knowledge, and therefore infer (predict) each other's next conversational move [5]. However, while verbal interaction certainly involves normative elements, they hardly help capturing its causal dynamics. A cognitive account has been proposed that sets dialog exchanges as the primary site of language experience [6]–[8]. A central tenet of this account is that successful dialogs depend on the fast interactive alignment of procedural and representational contents between interlocutors (see [6], p. 170). Notably, this would be achieved via “resource-free” processes such as priming. For example, as the conversation progresses each interactant will tend to use a common set of words, sentences, communicative styles, thereby establishing mutual resonance relationships at different levels of complexity [9].

Indeed, such perspective matches the intuition that when a conversation is successful we end up speaking “the same language” as our interlocutor. However, everyday conversations also implicate spontaneous adaptation to new, unpredicted discourse paths. For example, changes in one's own or other's beliefs or desires determine the introduction of a new topic or, at times, highlight the chance for a metaphoric or ironic statement [2], [10], [11]. We continuously make room for other minds [12] using our ability to “mentalize”, that is, to promptly and spontaneously attribute mental states such as beliefs and desires to the self and others [13].

Mentalizing is a core social skill operative since infancy in neurotypical development [14], [15]. Explicit reflection upon one's own and others' true or false beliefs emerges between the ages of 4 and 6 years, when children begin to distinguish between belief-based and reality-based thoughts [16]. Importantly enough, 5- and 6-year-old children become also faster in responding correctly than incorrectly to false-belief tasks, suggesting that decision-making skills develop along with mentalizing abilities [17]. From age 8–10 years onwards, children can master higher-order mentalizing activities, and conversation becomes a central focus of emotional and cognitive development [18].

However, in some cases the ability to mentalize is severely impaired. Individuals with Asperger Syndrome or High Functioning Autism (hereafter, HFA) are phenotypically characterized by a marked impairment in spontaneous mentalizing, while intelligence and formal language skills are preserved [19], [20]. Individuals with Asperger Syndrome/HFA can learn to handle conversational interaction as a task [21], [22]. However, explicit strategies are unlikely to compensate for the absence of spontaneous adaptation to dynamic changes. Consequently, social relation problems commonly surface in adolescents and young adult individuals with Asperger syndrome/HFA [23]. So far, the impact of mindblindness on the dynamics that characterize conversational behavior has not been investigated.

In this work, we captured conversational blueprints by resorting to human computational modeling. We designed a meaning-free, low-level acoustic serial generative framework, composed by a Gaussian mixture model (GMM) [24], followed by an observed influence model (OIM) at the top level [25]. OIMs are built upon Markov models, which offer a stochastic interpretation of time series, and thus are apt for the analysis and recognition of event sequences in speech recognition and natural language processing [26]. Interaction effects within each conversation were modeled assuming that the two speech streams were cooperative, binary (silence vs. speech) stochastic processes. Further, we posited that whenever a process changes its state, it injects a corresponding auto-transition state in the other process, forcing synchronization and creating novel low-level auditory segments termed Steady Conversational Periods (SCPs) [27]. SCPs permit the calculation of transition probabilities both intra- and inter-processes, thereby picturing the fast mutual effects of dialogic exchanges. The resulting influence matrix shows how much the state one participant is in at time 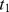 influences the state the other participant will be in at time 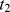 (inter-chain influence), as well as the how each participant proactively influences his/her own transition from state to state (intra-chain influence) [27], [28]. Using this approach, we aimed at verifying if the dynamics of flat (non arguing) dialogs depend on theory of mind abilities.

## Materials and Methods

### Participants

Data collection was run at the Scientific Institute “E.Medea” in S. Vito al Tagliamento (Pordenone, Italy). A young female psychologist acted as moderator in binary conversations with selected participants. The moderator was not aware of the study aims, clinical characteristics of the participant population, or individual clinical status (patient vs. control). She was introduced to all participants as a researcher who was interested in hearing their opinion on a set of topics.

Nine children and adolescents with a diagnosis of Asperger syndrome/HFA (8 males, 1 female, age range 7–14 years, mean  = 11) following the DSM IV (1994) criteria and the support of either the Childhood Autism Rating Scale (CARS, [29]) or the Autism Diagnostic Observation Schedule (ADOS, [30]) participated in the study. Nine gender- and age-matched typically developing peers were selected. All participants (N = 18) were made familiar with the moderator (acquaintance phase, about 10 minutes, not recorded). All individuals with Asperger syndrome/HFA had a clinical history of extensive rehabilitative training programs focused on limiting repetitive behaviors and enhancing social relationships in one-to-one and group interactions (range of program duration: 2–5 years). At the time of the experiment they were all still receiving rehabilitative training.

#### Ethics statement

Our research adheres to the basic ethical considerations for the protection of human participants in research according to the Declaration of Helsinki, and has been approved by the Ethics Committee of the Scientific Institute “E.Medea” (Bosisio Parini, Lecco, IT). The parents of potential participants first received a letter describing the study. Then a short phone colloquium cleared any remaining doubts. Both parents signed a written informed consent and at least one of them (or a caring relative, e.g. grandfather) accompanied the participant to take part in the experimental session. Children and adolescents were explicitly asked whether they would agree to talk with the moderator and exchange views on a set of familiar topics, which would later be analyzed by researchers. All participants gave their verbal consent.

#### Data collection

Participants entered an anecohic sound-proof room which contained two boots separated by a transparent glass pane. They sat on one side of the glass pane and were always in full visual contact with the moderator, who sat on the other side. Participants were offered puppets and toys to play out during the acquaintance phase. All participants as well as the moderator wore headphones and spoke to a calibrated recording microphone in order to output two synchronized but separate (unmixed) audio sources [27]. The headphones and microphone were explored as a playful practice. We adhered to a type of participatory research in which participants with and without disabilities are encouraged to actively contribute their views and find their own solution to establishing a pleasant interactive setting [31]. Children and adolescents were gradually involved in a dyadic conversation lasting about 10 minutes (experimental recording). It has been shown that topic change constitutes the second major type of variation in children's language samples [32]. To control for this issue, the moderator introduced five topics in a flexible sequence (school activities, hobbies, friends, food, family), prompting an active conversational exchange. The moderator was instructed to follow the participants' reasoning path and react appropriately, avoiding a rigid question-answer scheme. A total of 18 semi-structured conversational samples were collected. A speech/silence thresholding was performed on the raw signals, obtaining a signal 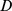, formed by two binary arrays 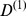 and 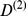, each one of length 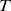, and each related to a particular interactant (participant/moderator). The percentage of speech and silence samples for the two classes are reported in Table 1, highlighting a similar profile.

**Table 1:** Average percentage of silence and speech samples in the conversations of the two classes of participants.

Class	speech	silence
Asperger/HFA	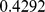	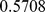
Controls	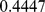	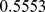

#### Neuropsychological profile

**Figure 1:**
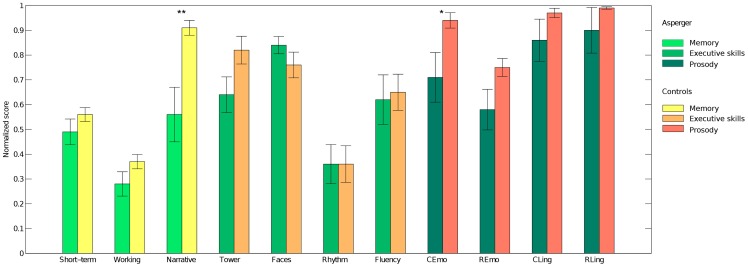
Neuropsychological profile. Legend: C/REmo  =  Emotional prosody comprehension/repetition; C/RLing  =  Linguistic prosody comprehension/repetition. Significance flags: **  = *p*<0.01; * =  *p*<0.05.

After the recording session, all participants received a neuropsychological screening targeting: a) linguistically mediated short-term/working memory and narrative memory; b) executive skills; 3) verbal prosodic abilities. The tests were: forward and backward digit span [33]; narrative memory, Tower of London and visual face recognition [34]; rhythm perception task (targeting the ability to reproduce rhythmical beat sequences [35]); verbal fluency [36]; comprehension and repetition of emotional (happy, sad, angry) and linguistic (affirmative, interrogative, imperative) prosodic contours [37]. Accuracy/performance data were normalized to max = 1 before entering statistical analyses (for the verbal fluency test, a global mean value was obtained by collapsing the number of uttered words across target letters F, A, S). Two-tailed t-tests were used to verify the presence of significant differences between the groups (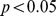). Fig. 1 displays the neuropsychological profile of Asperger syndrome/HFA and neurotypical control participants.

A significant difference was found for the narrative memory test (t(16)  = −3,057, p = 0.008), suggesting poor verbal long-term memory storage in the Asperger/HFA group. As expected, participants with Asperger syndrome/HFA showed difficulties in emotional prosody comprehension (t(16)  = −2,199, p = 0.043), and a tendency to significance was evident for emotional prosody repetition (t = −1,852, p = 0.083), and the Tower of London score (t = −1,909, p = 0.074), with participants with Asperger syndrome/HFA showing emotional repetition/planning difficulties. No significant differences were found in short-term/working memory, face recognition, linguistic prosody, rhythm perception and verbal fluency skills (all ps 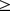
0.15).

### The observed influence model

**Figure 2:**
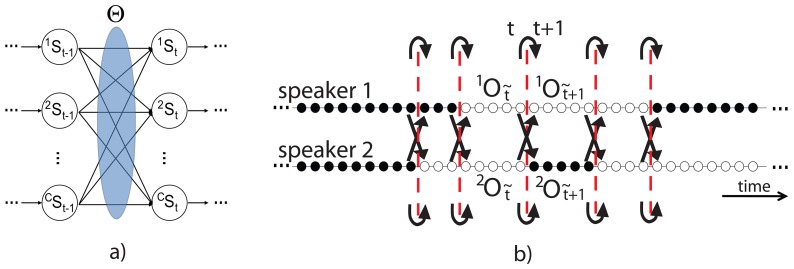
The generative framework. The Figure represents two main aspects: a) State factorization exploited in an observed influence model. The area named 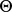 indicates the influence factors that apply to the state transitions, depicted as directed arrows. b) Synchronization through Steady Conversational Periods. There are two audio processes, 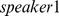 and 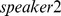, sampled at a given frequency, where audio samples are shown as *speech* (black dots) and *silence* (white dots) values. Continuous periods of speech or silence are not synchronized, so it is not possible to evaluate a first-order statistical transition probability among the periods. Global transitions (dashed red lines) define the SCPs, thus allowing the calculation of first-order transition probabilities (black arrows).

The observed influence model (OIM) is a simplified version of the influence model [25]; while OIM relies on interacting Markov chains, the influence model focuses on hidden Markov chains. We define the state variable of a Markov chain as 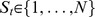, and 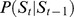 as the transition probability of a first-order Markov chain. OIM factorizes the multi-process conditional relations among 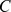 Markov chains by means of a weighted linear combination of pairwise *inter-chain* and *intra-chain* transition probabilities. Considering first-order Markov chains with N states, the (full) factorization of the multi-chain transition probability is:
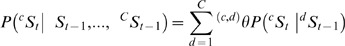
(1)with 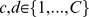, 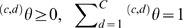. The value 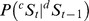 represents the probability of going from state 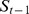 of the chain 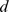 to state 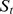 of the chain 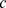. The weight 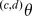 represents the influence that chain 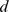 exerts on chain 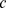. A sketch of the model is depicted in Fig. 2 a.

A first-order influence model is thus defined as 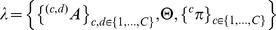, where 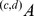 is the *intra* -chain transition matrix when 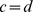, and represents the dynamics of a single process *per se*. When 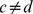, we consider the *inter* -chain matrices, modeling how much a state of a chain conditions the next state of the other chain. The 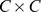 matrix 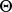 contains the influence weights, and 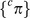 contains the (independent) initial probability distributions for all processes, *i.e.*, 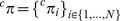, where 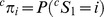.

The OIM transition factorization has space complexity 
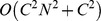
, where 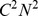 is due to the transition tables parameters, and 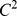 to the influence coefficients. OIM learning of the 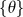 coefficients is performed by standard constrained gradient descent [24], [38], while the 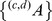, 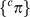 parameters are estimated by simple state counting.

#### The SCP model

Let us suppose to have a dialog with 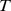 instants. Within the recording setting described above, a dialogue can be represented as an OIM, but the lack of effective synchronization between the start/end instants of the speech/silence periods leads to problems in evaluating inter-chain conditional dependencies.

Thus, we proposed to use a novel feature based on the core assumption that turn-taking dynamics are interactionally controlled [39], called Steady Conversational Period (SCP). SCPs are built on the duration of continuous slots of silence or speech [27]. The SCP extraction procedure assumes that whenever a process (i.e, silence or speech) changes its state, it causes a *global* transition that affects also the opposite process, inserting a novel auto-transition state (see the red dashed lines in Fig. 2 b). The fragmentation caused by global transitions synchronizes the processes, creating 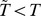 different SCPs 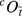, where the apex 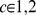 indexes the speaker and 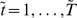 enumerates the different SCPs. The introduction of SCPs in the model makes it feasible to evaluate first-order intra- and inter-chain conditional probabilities (black solid line in Fig. 2 b).

In order to take into account the different durations of each silence and speech segment, all SCPs related to speech and silence were labelled as 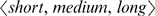, after a Gaussian clustering over a training dataset, performed with the Expectation Maximization algorithm [40].

More formally, given the clustering, each SCP 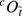 takes one label among 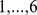, where 1,2,3 address short, medium and long continuous periods of speech, respectively, and the same applies with 4,5,6 for the silence periods. The number of states was decided as to maximize classification accuracies (see Table 2). It is worth noticing that the obtained performances were similar if 4 states were chosen (short and long periods), while suboptimal results were obtained with only 2 states, and more than 6. This quantization gives rise to *quantized* SCPs sequences 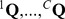; pooled together, these sequences form a dialog 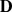.

**Table 2:** Cluster boundaries (in seconds) for the SCP durations, for the speech and silence SCP typology.

SCP type	short	medium	long
silence	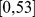	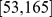	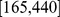
speech	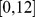	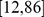	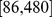

After that, an observed influence model 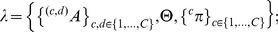 was fitted to a dialog 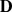.

The intra-chain parameters of the model intuitively indicate the conversational trend of each participant considered separately. The inter-chain transition parameters encode first-order state dependencies among processes, and influence factors mirror the influence that a process exerts on the other, independently on the state assumed by the single processes.

A classification involving the OIM has to be carried out considering carefully the order with which the observation sequences are organized. For example, within a dyad, in which the second process/speaker exerts a strong influence on the first one, a model is learnt where the weight 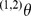 is high. In order to recognize such situation in a classification scenario, the relative ordering of the sequences has to be preserved, *i.e.*, the second sequence has to be the one related to the process that influences the opposite one. If this cannot be ensured, a reasonable strategy for extracting the “correct” classification score would be the following: the dialogs 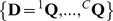 are presented to the model in all their possible orderings (having diads, only two), indexed by 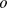, collecting all corresponding likelihood scores 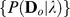. The correct likelihood score would thus be the highest one.

#### The generative score space

In order to increase the classification accuracy of the generative framework, and, at the same time, get an insight on how the model works in encoding the dialogs, we built a generative score space 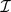. Following [41], a generative score space can be built by considering each dialog 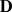 as completely represented by a generative model 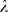 with its parameters, which was trained on the dialog. Formally, the observed dialog 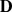 is mapped through 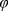 into a fixed-length score vector 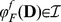,
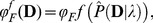
(2)where 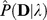 is the set of distributions that define a generative model, 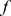 is a function of these distributions and 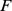 is some operator applied afterwards. For instance, in case of the Fisher score [42], 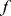 is the log likelihood, and the operator 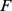 produces the first-order derivatives with respect to the parameters. Another example is the TOP kernel [43] for which the function 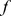 is the posterior log-odds and 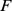 is still the gradient operator.

In these cases, the generative score-space approaches help to distill the relationship between a model parameter contained in 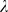 and a particular data sample modeled by that parameter. After the mapping, a score-space metric must be defined in order to calculate the distances in that space.

In our case, 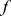 was the parameter extractor function (*i.e.*, the function that estimates the parameters of a statistical distribution), 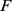 the identity operator, and as a metric we selected the Euclidean one. In synthesis, 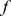 extracted the transition parameters (by simple counting) and the influence coefficients (by gradient descent).

Given a set of 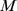 classes of dialogs, each formed by 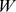 sequences, the space 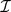, could be seen as formed by a set of multidimensional class-labeled samples; actually, on each sequence, a model is trained, that provides a set of features/parameters. Therefore, standard tools of data analysis can be applied. We wanted to highlight the discriminative power of the features in a classification context, and therefore we applied a feature selection (or ranking) strategy, and, subsequently, we applied different discriminative classifiers on the feature subset. The feature selection/ranking strategies together with the discriminative classifiers employed will be detailed in the next section. Discriminative classifiers were preferred, because they directly focus on estimating class posterior probabilities instead of modeling class distributions. Such classifiers should also be less affected by the dimensionality problem.

Encapsulating OIMs in the SCP-based generative framework is straightforward. The embedding in 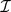 produces an ensemble of features 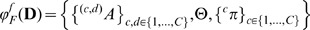, for each dialog: considering that the space complexity of the whole model is 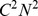 (the transition matrices) 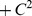 (the influence coefficients) 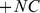 (the initial distributions), and fixing 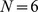 (corresponding to 3 states for the speech duration and 3 for the silence duration) and 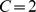 (two speakers), we obtained 160 values. This parameter setting was used in all the reported experiments.

The rationale underlying the choice of this score space is that by employing parameters as features, and analysing the features with feature selection strategies, we can understand which portions of a model are more effective in capturing the unique characteristics of the classes. For example, capturing the fact that a particular state transition is strongly discriminant for a certain class implies that such transition is peculiar for that model. This property cannot be mimicked by Fisher score based approaches, where the basic tool is the differentiation with respect to particular quantities (i.e., the log-likelihood in the Fisher score), which can suffer of the so-called “wrap-around” problem, where very different data points may map to the same derivative (see [44] for an example).

## Results

As mentioned, for each of the 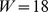 participants we learned an individual dialog model, giving rise to 160 parameter values (having 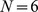, 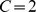) per participant; in the following we will use the term “feature” as a synonym of parameter.

The point was to understand how well the two classes of participants could be separated, i.e., how different the parameters of the two classes were. To this aim, we adopted a classification framework, in which one of the participants (the *test* participant) has to be classified in one of the two classes, considering the similarity between his/her model parameters (i.e., features) and the ones of the remaining participants (the *training* participants).

We applied the classification procedure considering iteratively each of the 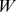 participants as the test element, keeping the remaining ones as training. This strategy is termed *cross-validation* since it validates the classification performance shuffling the elements that are used to represent (or train) a class and the ones employed as test. This particular cross-validation strategy is dubbed *leave-one-out* (LOO) since, in turns, a single element is assumed as test element. Its use is particularly suited for limited samples.

Due to the high number of parameters, and for the purpose of understanding intuitively in which sense the two classes differ, we used a feature selection strategy. This allowed us to find out which one of the 160 parameters was most important for class separation; in other words, we highlighted the parameters that are most discriminant.

Feature selection is an open research field of pattern recognition, and many policies have been designed so far. One of the most widely employed is based on the concept of stability [45]. In simple terms, at each iteration of the LOO strategy a pool of features is selected as the most informative (*i.e.*, giving rise to a high classification performance) employing a particular feature selection approach, namely, the forward feature selection strategy [24]; if this set is consistent across different iterations of the cross-validation strategy, then it is considered to be stable, i.e. invariant to the nature of the training and the testing set.

We employed a slightly different approach, as more apt to deal with different feature selection policies. By fixing 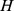 feature selection policies, and for each policy a cross-validation LOO strategy, we evaluated how many times a feature is selected as discriminant for that particular policy, considering at the end all the different policies for choosing the final subset. As a classifier for the feature selection step we used the K-nearest neighbor classifier (K = 3) [24].

In our case, we employed 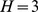 different policies of feature selection, that is, forward feature selection [46], branch and bound [47], and min-redundancy max-relevance [48]. Each policy may provide a subset of 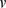 best features. In our case, we defined 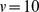.

At each iteration of the cross-validation of a single policy, an instance of 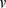 features is produced. With 18 participants, this corresponds to 18 instances. Considering the three policies of feature selection, 64 instances were obtained. After that, each of the 160 features was evaluated, counting how many times it appeared in an instance, thus assigning a frequency score to each feature. The scored features were then ordered (see Table 3, where 10 features are reported); together with the number of the features, we report their functional significance, i.e., the parameters they represent. These features can be thought of as independent on the particular strategy of feature selection. Manifestly, only transition parameters are present, while influence coefficients and initial probabilities are not present.

**Table 3:** Ordered features: each feature is ordered based on its frequency in the pool of features selected by the 3 feature selection strategies.

Feature	Frequency	Parameter
**17**	0.83	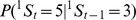
**80**	0.46	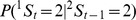
**82**	0.36	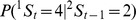
**89**	0.32	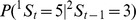
**15**	0.3	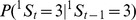
**107**	0.3	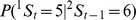
**53**	0.24	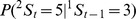
**38**	0.21	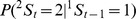
**14**	0.15	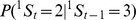
**55**	0.14	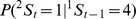

On the right, the corresponding parameter for each feature. For the sake of clarity, only 10 features are reported.

**Figure 3:**
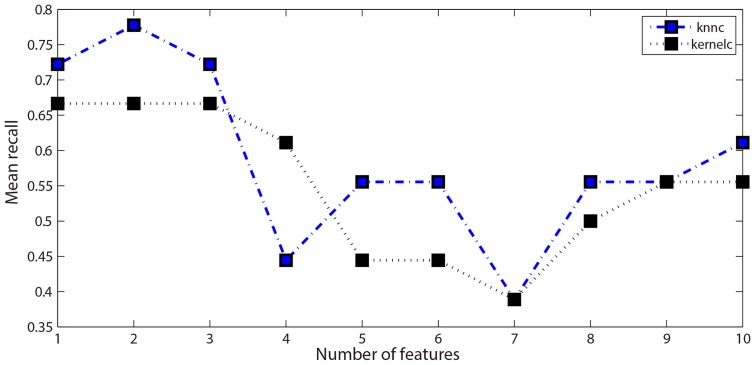
Feature analysis. Classification accuracies (measured in term of mean recall of the two classes) depending on the number of features employed, where the features are considered in order of importance (see Table 3).

At this point we evaluated which subset ensured maximal classification accuracy. We thus considered one feature at a time, starting from the most frequent one, feeding two different classifiers and evaluating the LOO classification accuracy. As classifiers, we considered the K-nearest neighbor classifier (K = 3) and the linear Support Vector Machine, both implemented using the MATLAB Prtools [49]. The classification performances are reported in Fig. 3, in relation to the number of features employed.

The curves indicate that the first 2 features are the most important for the classification. They represent the probability of the moderator to speak for a long time after a medium silence interval (feature 17), and the probability of the moderator to speak for a medium interval after the participant (Asperger/HFA or control) has spoken for a medium-length segment (feature 80). Keeping these two features, the LOO classification performances are detailed in Table 4.

**Table 4:** Leave-One-Out classification performances, considering only the best features 17 and 80.

Classifier	Asperger		Controls	
	Precision	Recall	Precision	Recall
knnc	0.69	1	1	0.56
kernelc	0.64	0.78	0.71	0.56

**Figure 4:**
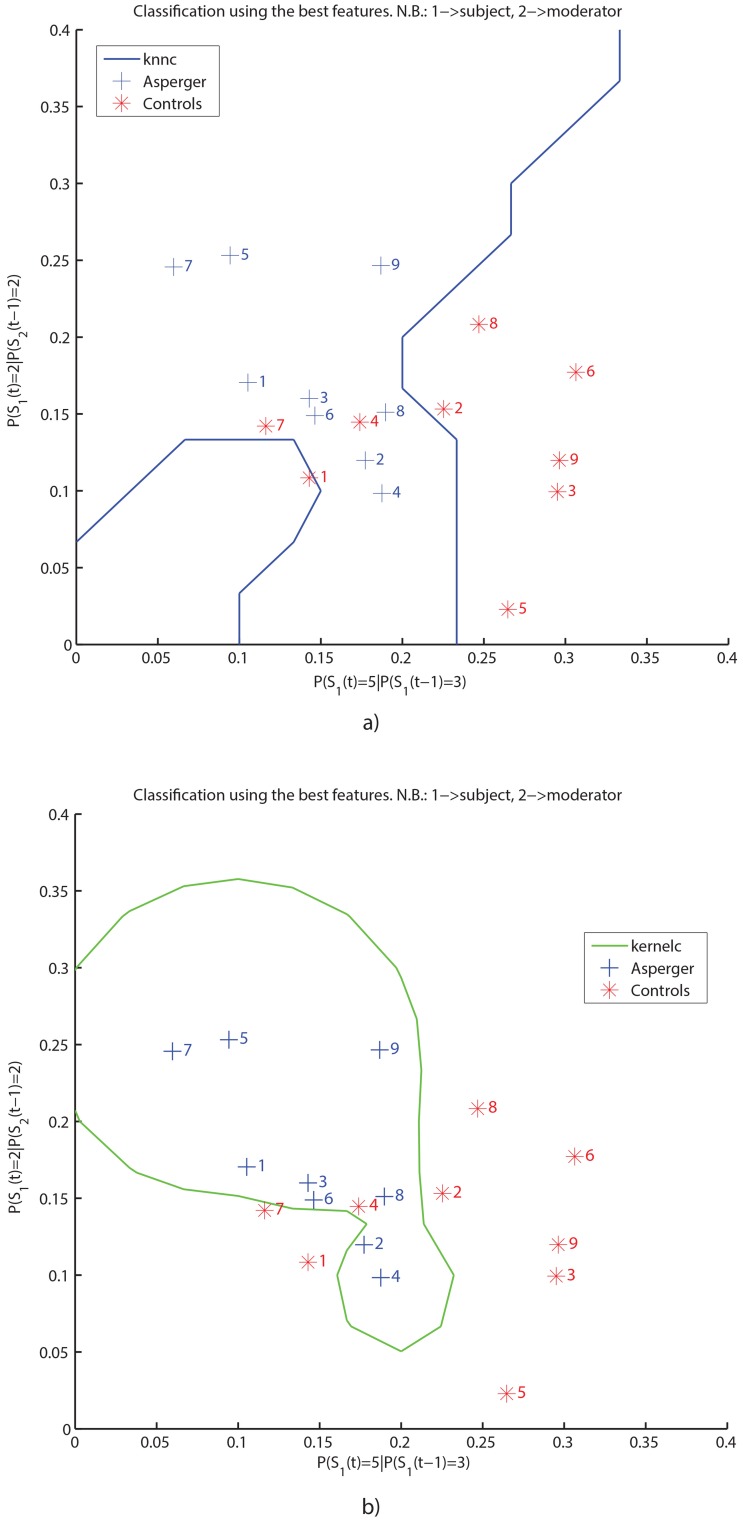
The generative score space. Separation boundaries in the generative score space with a) the K-nearest neighbor classifier and b) the Support Vector Machine classifier (the kernelc implementation of Prtools), both performed on the best features found by feature selection.

In addition, we report the values of the two features for all the participants in a 2D space (see Fig. 4), showing also the classification boundaries identified by the two different classifiers: boundaries are obtained by opportunely sampling the feature space, assigning a label to each point, and defining a boundary where the classification labels change from one class to the other. Employing the K-nearest neighbor classifier (knnc) and the linear support vector machine (kernelc) we obtained the separation accuracies, in terms of precision and recall, reported in Table 5. These values reflect the degree of certainty (max = 1) with which the two classes can be separated based on the selected features.

**Table 5:** Separation performances, considering only the best features 17 and 80.

Classifier	Asperger		Controls	
	Precision	Recall	Precision	Recall
knnc	0.81	1	1	0.78
kernelc	0.9	1	1	0.89

To increase the reliability of our findings, we replicated the classification procedure in a subsample of our data. Participants with Asperger syndrome/HFA number 5 and number 7 had the lowest scores across groups on the narrative memory test (0.03 and 0.06, respectively) and emotional prosody comprehension (0.33, 0.08). To investigate if automatic classification was influenced by neuropsychological differences in single participants, we first we re-run the statistical comparisons on the mentioned tests without Asperger syndrome/HFA participants 5 and 7, and the corresponding age-matched control individuals. We still found a significant difference for the narrative memory test (t(12)  = −2.541, p = 0.026), but no difference in emotional prosody comprehension (t(12)  = −1.448, p = 0.173). We then highlighted the position occupied by each participant in the classifier space, according to each feature. At visual inspection, Fig. 4 suggest that participants 5 and 7 with Asperger syndrome/HFA cluster with patients 1 and 9, and control participants 9, 8, 6, and 3 form a separate cluster of their own. This observation deems unlikely that specific problems with emotional prosody recognition or complex language memory tasks in participants 5 and 7 with Asperger syndrome/HFA would drive the classification effects. To verify this point, we excluded participants 5 and 7 from both groups and re-run the classification procedure using the K-nearest neighbor classifier; surprisingly, the classification performances were almost the same than considering the whole sample population, that is: Asperger, Precision  = 0.7, Recall  = 1; Controls, Precision  = 1.0, Recall  = 0.57. For a comparison, see the LOO classification results of Table 4.

## Discussion

We used pattern recognition as a lens into meaning-free conversational blueprints [50]. We were able to model the mutual effects of dialogic exchanges by forcing synchronization of silence/speech sequences (Steady Conversational Periods, SCPs). By analyzing both intra- and inter-processes (speech/silence) transition probabilities, the conversations of individuals with Asperger syndrome/HFA were reliably and automatically distinguished from those of gender- and age-matched peers. The synchronization assumption was sufficient to tear apart the clinical from the non-clinical group without relying on any higher-order feature (e.g., meaning, word frequency, syntactic complexity).

Previous work showed the effectiveness of such a novel approach on groups with either extremely different speech rates (e.g., children vs. adults) or speech modes (e.g., flat conversations vs. lively discussions) [27], [28]. We now classified two samples with a similar speech rate, similar age and a compatible higher-order cognitive profile, but crucially different in the spontaneous attribution of mental states to the self and others [18]–[20], [23]. It follows that theory of mind skills exert a sizable influence onto basic speech initiative behavior.

This result is in line with theories that try to capture the biological bases of human conversational talent as stemming from the coordination of speech, gestures, kinematics and sensory attention [2]. We suggest that across-level interactant synchronization might be a key concept in investigating conversational speech dynamics [50], thereby extending the entrainment approach beyond purely linguistic representations [6].

The creation of the SCPs as a relevant index is motivated by several reasons, not restricted to a mere algebraic point of view or to the assumption of synchronicity. At a basic level, they partially reflect the respiratory kinematics which co-determine the dynamics of self-initiated speech [51], [52]. Such kinematics provide the basis for the coordination of prosodic and syntactic planning [53]. SCPs might thus reflect a significant subset of the variance characterizing the real-time interplay of physiological, neuropsychological and intentional factors which determine the dynamics of speech alternation in a dialog, including turn-taking strategies usually negotiated via audiovisual intentional cues [54], [55].

The crucial point of our work was to obtain a successful classification. However, the extracted features also provide some insights into the underlying processes (see Fig. 4). For example, the moderator displayed a tendency to self-influence the change between silence and speech while conversing with control participants, suggesting a more directive role. Instead, consistent instances of speech activity on the part of individuals with Asperger syndrome/HFA determined segments of silence on the part of the moderator, suggesting that what the children said, or the way they said it, interrupted the flow of the verbal exchange. The fact that in our analysis we abstracted away from meaning must not be taken to imply that meaning has no influence, but simply that SCPs highlight the reverberations of meaningful or less meaningful verbal exchanges on lower functional levels. It is likely that with larger group samples the interactions can be more finely pictured by more significant features.

We did not test theory of mind skills directly because all patients had already been exposed to similar tests many times during clinical assessment and rehabilitative training. We could document the correlated difficulty in emotional recognition using a novel test setting ([37], see Fig. 1). We also found a significant difference in narrative memory between Asperger/HFA and neurotypically developing participants. Recalling complex verbal material relies on inferential bridges that the listener must make to obtain a coherent picture of the different characters. As the spontaneous attribution of mental states is a key stage in this process, individuals with Asperger syndrome/HFA are likely to fail in tasks requiring the retrieval of a coherent story [56]. Finally, a recent work suggests that Asperger syndrome/HFA individuals may use inner speech for short-term/working memory tasks, as control peers do, but not for planning [57], a fact that could explain the planning difficulties in our group of patients.

Human beings might use forms of entrainment via resource-free processes such as priming to share information among each other and within themselves across functional levels. Conversational entrainment need not be representational in format, but can determine the game of parts of influencing each other's next move [50]. This perspective is not in contradiction with the idea that we can predict or infer the interlocutor's possible next move based on general cooperation assumptions [5]. Simply, rational inference now becomes a local-value strategy, capturing the mechanics of some single frames within the dynamic, effortless and kaleidoscopic flow of conversational speech, which we just began to tackle. For example, much research is needed on how the brain effectively manages the inherent complexity that our analysis highlighted [58]. From a neurocognitive viewpoint, the concept of mutual knowledge implies that information from multiple sources must be at the same time flexibly integrated within an individual's perceptual focus [59], [60], as well as shared with the interlocutor. Future research will tell us how much the brain synchronizes to the pleasure of a chat.
